# The epidemic volatility index, a novel early warning tool for identifying new waves in an epidemic

**DOI:** 10.1038/s41598-021-02622-3

**Published:** 2021-12-10

**Authors:** Polychronis Kostoulas, Eletherios Meletis, Konstantinos Pateras, Paolo Eusebi, Theodoros Kostoulas, Luis Furuya-Kanamori, Niko Speybroeck, Matthew Denwood, Suhail A. R. Doi, Christian L. Althaus, Carsten Kirkeby, Pejman Rohani, Navneet K. Dhand, José L. Peñalvo, Lehana Thabane, Slimane BenMiled, Hamid Sharifi, Stephen D. Walter

**Affiliations:** 1 Faculty of Public Health, University of Thessaly, Thessaly, Greece; 2 Department of Medicine and Surgery, University of Perugia, Perugia, Italy; 3 Department of Information and Communication Systems Engineering, University of the Aegean, Aegean, Greece; 4 UQ Centre for Clinical Research, Faculty of Medicine, The University of Queensland, Herston, Australia; 5 Research Institute of Health and Society (IRSS), Université Catholique de Louvain, 1200, Brussels, Belgium; 6 Department of Veterinary and Animal Sciences, University of Copenhagen, Copenhagen, Denmark; 7 Department of Population Medicine, College of Medicine, QU Health, Qatar University, Doha, Qatar; 8 Institute of Social and Preventive Medicine, University of Bern, Bern, Switzerland; 9 Odum School of Ecology, University of Georgia, Athens, GA 30602 USA; 10 Sydney School of Veterinary Science, The University of Sydney, Camden, NSW Australia; 11 Unit of Noncommunicable Diseases, Department of Public Health, Institute of Tropical Medicine, Antwerp, Belgium; 12 Department of Health Research Methods, Evidence, and Impact, McMaster University, Hamilton, ON Canada; 13 Pasteur Institute, University of Tunis El Manar, Tunis, Tunisia; 14 HIV/STI Surveillance Research Center, and WHO Collaborating Center for HIV Surveillance, Institute for Futures Studies in Health, Kerman University of Medical Sciences, Kerman, Iran

**Keywords:** Disease prevention, Public health

## Abstract

Early warning tools are crucial for the timely application of intervention strategies and the mitigation of the adverse health, social and economic effects associated with outbreaks of epidemic potential such as COVID-19. This paper introduces, the Epidemic Volatility Index (EVI), a new, conceptually simple, early warning tool for oncoming epidemic waves. EVI is based on the volatility of newly reported cases per unit of time, ideally per day, and issues an early warning when the volatility change rate exceeds a threshold. Data on the daily confirmed cases of COVID-19 are used to demonstrate the use of EVI. Results from the COVID-19 epidemic in Italy and New York State are presented here, based on the number of confirmed cases of COVID-19, from January 22, 2020, until April 13, 2021. Live daily updated predictions for all world countries and each of the United States of America are publicly available online. For Italy, the overall sensitivity for EVI was 0.82 (95% Confidence Intervals: 0.75; 0.89) and the specificity was 0.91 (0.88; 0.94). For New York, the corresponding values were 0.55 (0.47; 0.64) and 0.88 (0.84; 0.91). Consecutive issuance of early warnings is a strong indicator of main epidemic waves in any country or state. EVI’s application to data from the current COVID-19 pandemic revealed a consistent and stable performance in terms of detecting new waves. The application of EVI to other epidemics and syndromic surveillance tasks in combination with existing early warning systems will enhance our ability to act swiftly and thereby enhance containment of outbreaks.

## Introduction

Early warning tools are crucial for the timely application of intervention strategies and the mitigation of adverse health, social and economic effects associated with epidemics. Sentinel networks in combination with information technology infrastructures in public health^1^ provide data for the detection of spatial and temporal aberrations in the expected number of cases for groups of clinical signs and symptoms^2^. Several modelling frameworks exist for the analysis of such data. For example, the moving epidemic method is used to monitor, among others, the start of flu epidemics^3^. Further, methods based on seasonality patterns, the link between pathogens and meteorological parameters^4^ and/or the measurement of vector indices for vector-borne pathogens^5^ are also available.

Once an epidemic erupts, growth models can be used to predict the course of the outbreak and quantify its consequences. The advantages and limitations of these methods have been extensively discussed^6^. Machine learning algorithms have also been utilized with the most recent application being in the current COVID-19 pandemic^7^. Correlating the number of COVID-19 cases with parameters obtained using “big data” approaches can predict future rises in case numbers. For example, monitoring of digital data streams can provide an early indication of a rise in the COVID-19 cases and deaths within the subsequent two to three weeks^8^. All models have limitations arising from the imperfect nature of available data. The need for open, better, detailed data is imperative for the deployment of models with improved accuracy, better predictive ability, and therefore enhanced utility for the timely application of appropriate control measures for the COVID-19 pandemic^9^.

Our work introduces the Epidemic Volatility Index (EVI), which is inspired by the use of volatility indices in the stock market^10,11^. EVI is based on the moving standard deviation of the newly reported cases during an epidemic. First we present the rationale of EVI and then provide an example application with COVID-19 data from Italy and New York. Daily updated predictions—with a 48-h lag for confirmation purposes—are available online (http://83.212.174.99:3838) for all world countries and each of the United States of America. Results revealed a firm and consistent ability of EVI to predict the main COVID-19 epidemic waves, in all instances.

## Materials and methods

### The epidemic volatility index

**Figure 1:**
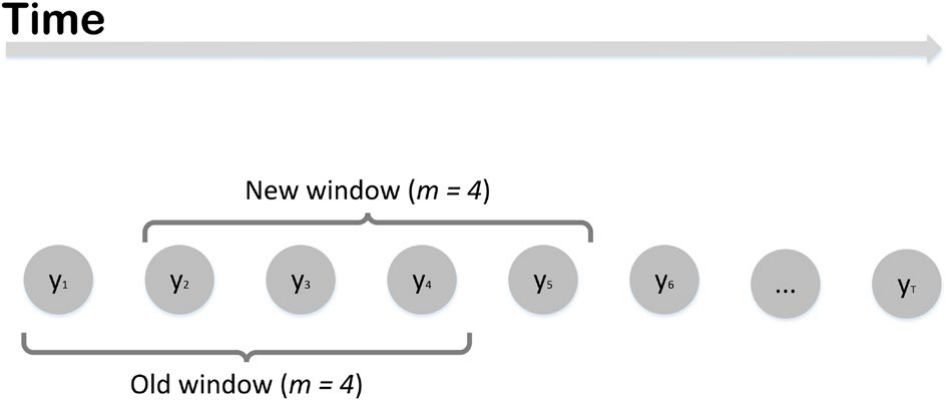
A time series $\left( {{\text{y}}_{1} ,{\text{y}}_{2} , \ldots ,{\text{y}}_{{\text{T}}} } \right)$ of epidemic data with an example rolling window of size ${\text{m}} = 4$. Rolling window for EVI is not fixed and is selected at each time point to achieve optimal performance.

EVI is based on the calculation of the rolling standard deviation for a time series of epidemic data (i.e. the number of new cases per day). The number of consecutive observations used for this calculation is the rolling window size-*m*. At each time step, for a rolling window of size *m*, the observations within the window are obtained by shifting the window forward, over the time series data, one observation at a time (Fig. 1).

For each rolling window the standard deviation of the newly reported cases is then calculated, allowing EVI to be estimated as the relative change of the standard deviation between two consecutive rolling windows. A warning signal is issued if (i) this relative change exceeds a threshold $c \left( {c \in \left[ {0,1} \right]} \right)$ and (ii) the observed cases at the current time point are higher than the average of the reported cases in the previous week.

### Criterion and desired accuracy

The accuracy of EVI is measured by its sensitivity $\left( {Se} \right)$ (i.e., the probability of correctly issuing an early warning for an upcoming epidemic wave) and its specificity $\left( {Sp} \right)$ (i.e., the probability of not signaling an alarm in the absence of upcoming waves) and depends on the criterion used to define what constitutes a noteworthy rise in the expected number of cases that is indicative of an upcoming epidemic wave. For example, a criterion can be, as in the example application that follows, a rise in the mean number of cases between two consecutive weeks higher than 20%.

For a specified criterion, the accuracy of EVI depends on the window size *m* and the threshold *c*, which should be selected in a way that achieves a desired accuracy target. One option is the selection of *m* and *c* values that lead to the best *Se* and *Sp* combination for EVI, through the maximization of the Youden index $\left( {J = Se + Sp - 1} \right)$^12^ and, hence, the overall minimization of false results (i.e., the total number of false positive and false negative early warnings). Another approach could be to select $m$ and $c$ such that the highest $Se \left( {or\;Sp} \right)$ is achieved with $Sp \left( {or\;Se} \right) = 1$ or not dropping below a critical value (e.g. 0.95). Advanced Receiver Operating Characteristic curve analysis can also be performed^13^ and selection of critical values can be based on indices that quantify the relative cost of false positive (i.e., falsely predicting an upcoming epidemic wave) to false negative (i.e., failing to predict an upcoming epidemic wave) warnings, like the misclassification cost term.

### Selection of optimal *m* and *c* and generation of an early warning

For a specified criterion and a desired accuracy target the optimal $m$ and $c$ are selected through an iterative process. Briefly, every time a new time point $t$ is observed:

1. Cases up to $t$ are analyzed for all possible window sizes $\left( m \right)$ and thresholds $\left( c \right)$.
2. For each of the $m$ and $c$ combinations, the $Se_{{t_{m,c} }}$ and $Sp_{{t_{m,c} }}$ are estimated for the specified criterion.
3. The $m^{\prime}$ and $c^{\prime}$ that give the best $Se_{{t_{m^{\prime},c^{\prime}} }}$ and $Sp_{{t_{m^{\prime},c^{\prime}} }}$ combination are selected (i.e., overall minimization of false results).
4. Based on $m^{\prime}$ and $c^{\prime}$, EVI is calculated at the new time point $t$ and a decision is made on whether a warning signal is issued or not.

**Figure 2:**
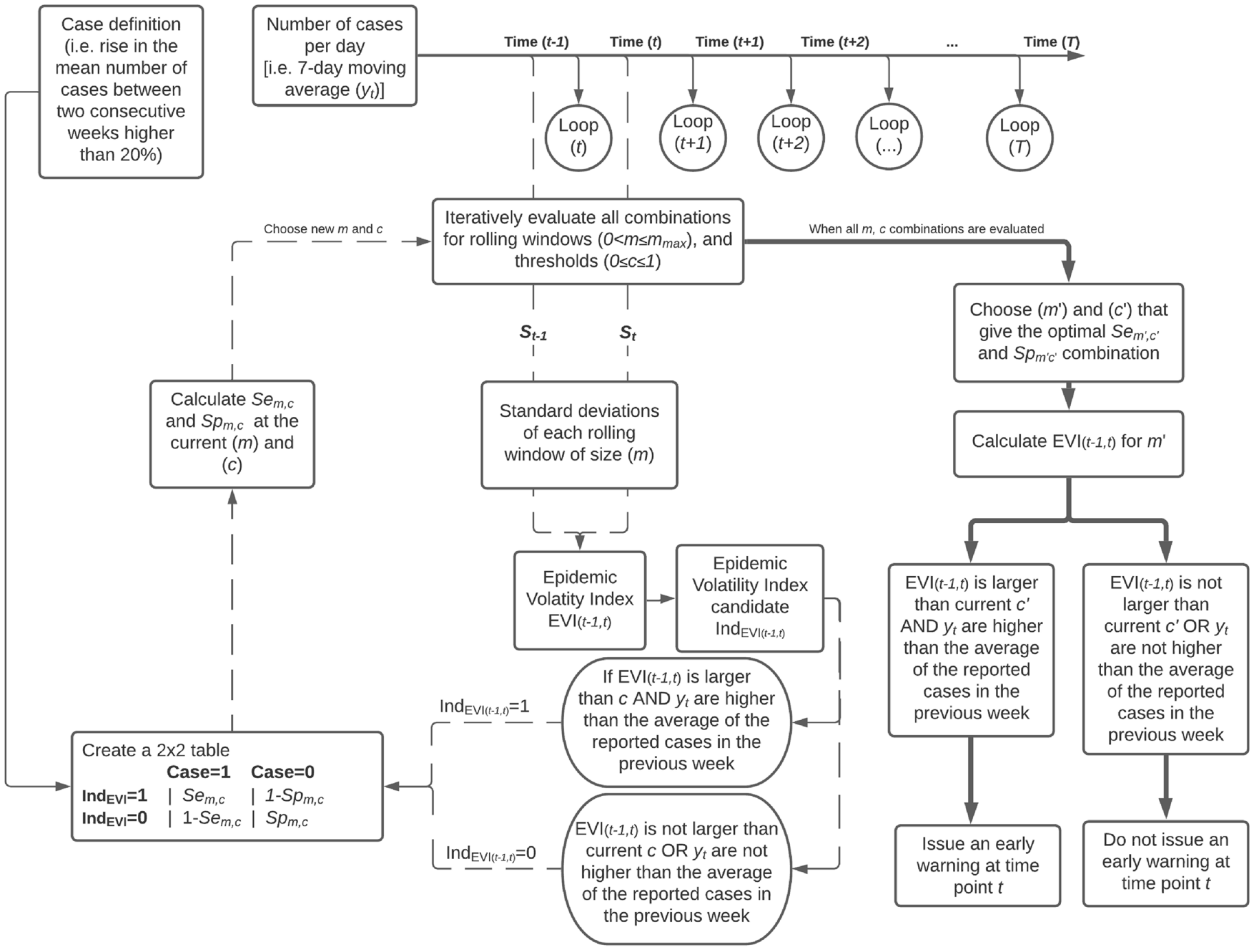
Graphical representation of the Epidemic Volatility Index (EVI) model. $T$ denotes the end of the time series, $t$ a time point of the time-series. $Se$ and $Sp$ stand for the sensitivity and specificity of the testing procedure calculated at each step of the algorithm. Solid lines are explanatory; at each time point dashed lines represent the iterative optimization process while the bold solid line denotes the end of the algorithm.

The graphical representation of the entire process is given in Fig. 2, while the statistical details are described in the “Appendix”.

### Overall accuracy and predictive values

It is possible, at each time point *t*, to calculate the positive and negative predictive values, defined as the probability of observing a rise or drop in the future number of cases, given that an early warning was issued or not, respectively. Finally, once the entire time series data has been observed the overall *Se*_*EVI*_ and *Sp*_*EVI*_ can be estimated.

### Sensitivity analysis

For each epidemic, the accuracy of EVI depends on the specified criterion. Ideally, different criterion values should be explored to identify which are suitable for the optimal monitoring of the epidemic. In the following example, sensitivity analysis based on an alternative criterion was performed.

### Example application

The current most serious threat to global health and economy^14^ is the COVID-19 pandemic that was first reported to the WHO China Country Office on December 31, 2019^15^. Data on the confirmed cases of COVID-19 were retrieved from the COVID-19 Data Repository, which is maintained by the Center for Systems Science and Engineering (CSSE) at Johns Hopkins University^16^. The number of daily confirmed new cases of COVID-19, for each country, from January 22, 2020, until April 13, 2021, were analyzed. Due to unnatural variability in the reported cases between working days and weekends, a 7-day moving average rather than the actual observed cases were analyzed. For the analysis, $m_{max}$ was restricted to 30 days in order to avoid the effect of potentially higher volatility from previous epidemic waves on the volatility estimates of the most recent data and the predictive ability of EVI for upcoming and perhaps milder epidemic waves.

The criterion used was an increase in the mean of expected cases, between two consecutive weeks, equal or higher than twenty percent. For sensitivity analysis, the detection of an increase in the mean of expected cases equal or higher than 50 percent was considered. Data were analyzed separately for each country and for each of the states of the United States of America that had experienced a total number of cases higher than 20.000, until April 13, 2021.

### Statistical software

All models were run in R^17^. The packages readxl^18^, ggplot2^19^, cowplot^18,20^ and readr^21^ were used. EVI is also available as a Stata module (type “scc install evi” in the command line)^22^ and as an R-package (https://github.com/ku-awdc/EVI).

### Results

Results for Italy, one of the most severely affected EU countries^23^, and New York, which was in the epicenter of the pandemic in the United States^24^, are presented in the main manuscript. Daily updated results for all world countries and each of the United States are available online at http://83.212.174.99:3838.

**Figure 3:**
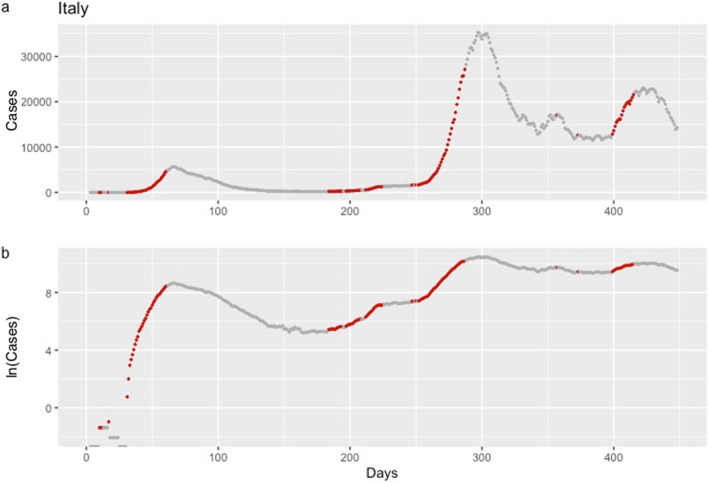
Daily confirmed cases of COVID-19 in Italy, from January 22, 2020, until April 13, 2021. Analysis is based on the criterion aiming to detect an increase in the mean of expected cases equal or higher than 20%. Red dots correspond to dates that, according to the Epidemic Volatility Index (EVI), an early warning was issued indicating that a rise in the COVID-19 cases is expected. Data are presented on the original scale (**1a**) and the logarithmic scale (**1b**), which facilitates the comparison of the steepness of the epidemic curve between the different waves.

**Figure 4:**
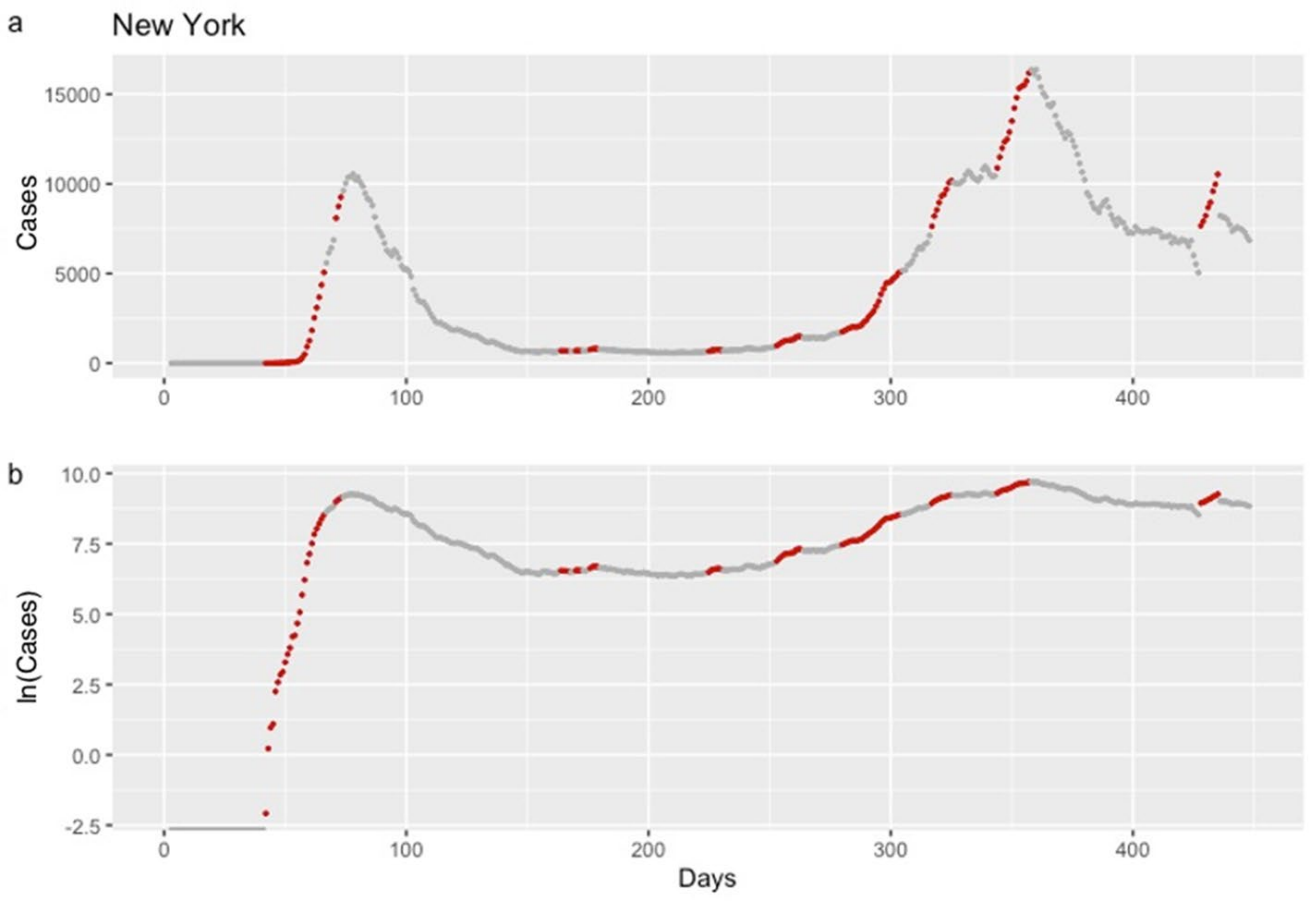
Daily confirmed cases of COVID-19 in New York, from January 22, 2020, until April 13, 2021. Analysis is based on the criterion aiming to detect an increase in the mean of expected cases equal or higher than 20%. Red dots correspond to dates that, according to EVI, an early warning was issued indicating that a rise in the COVID-19 cases is expected. Data are presented on the original scale (**1a**) and the logarithmic scale (**1b**), which facilitates the comparison of the steepness of the epidemic curve between the different waves.

**Figure 5:**
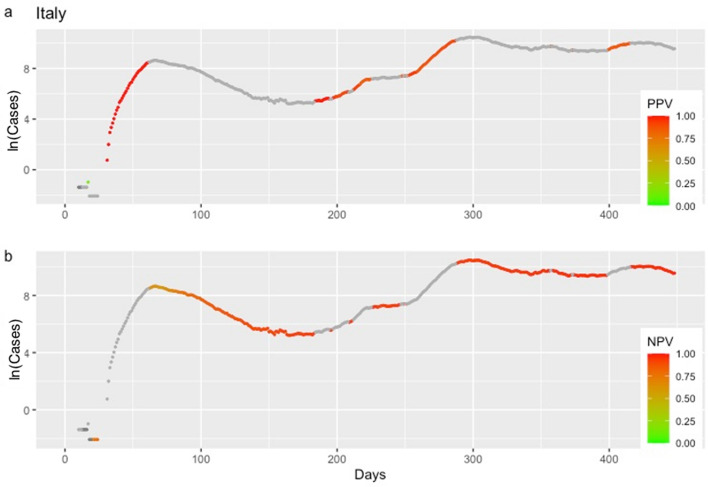
Positive and negative predictive values (PPV in **3a** and NPV in **3b**), for Italy, depending on whether or not an early warning was issued. Higher color intensity corresponds to predictive values closer to the value of 1.

**Figure 6:**
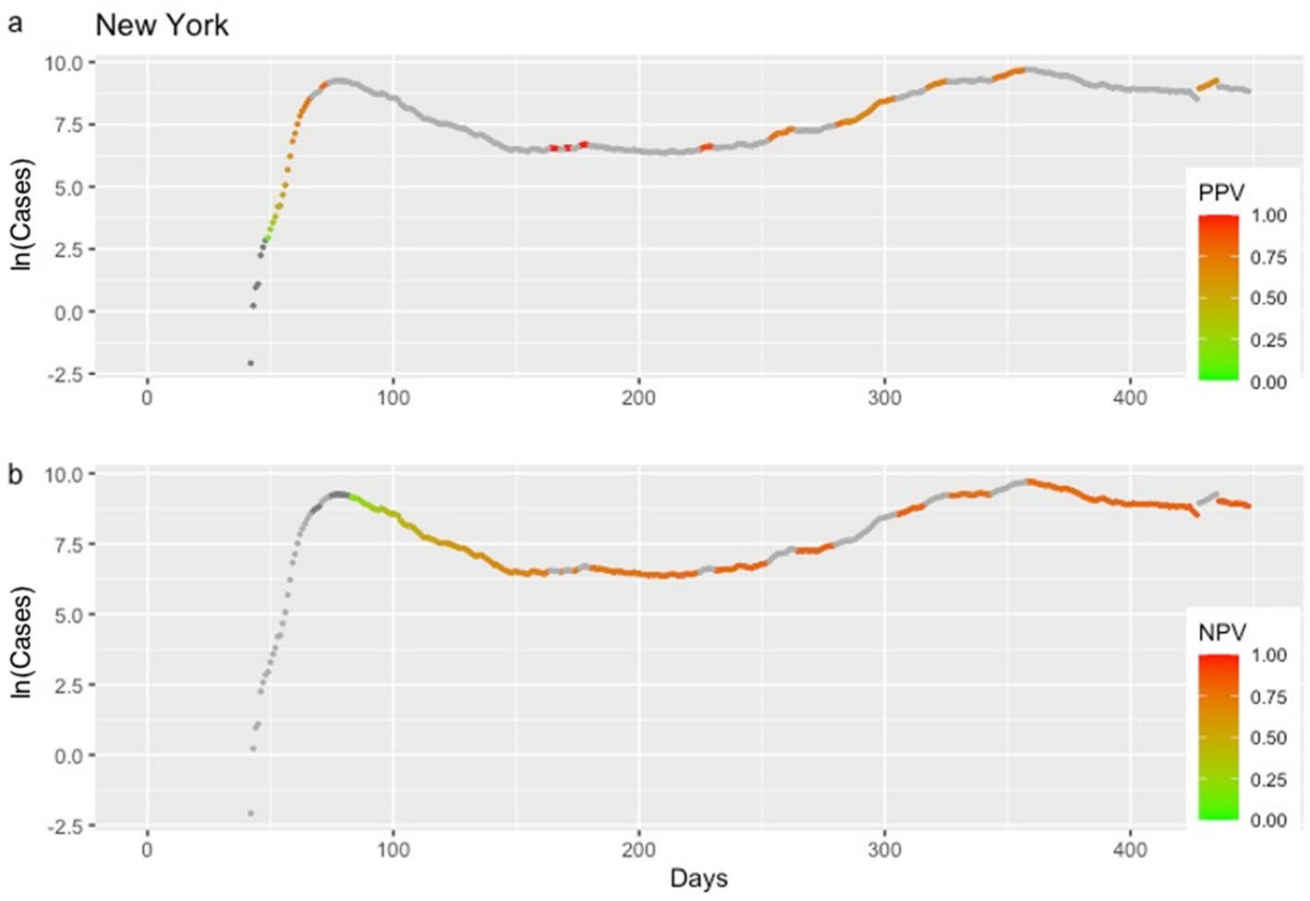
Positive and negative predictive values (PPV in **4a** and NPV in **4b**), for the state of New York, depending on whether or not an early warning was issued. Higher color intensity corresponds to predictive values closer to the value of 1.

Confirmed COVID-19 cases for Italy and New York State, from January 22, 2020, until April 13, 2021, are in Figs. 3 and 4, respectively. Red dots correspond to time points when an early warning was issued and indicate that, according to the defined criterion, an increase in the mean of expected cases equal or higher to twenty percent is expected in the coming week. Grey dots correspond to time points without an early warning indication. Further, positive and negative predictive values at each time point are in Figs. 5 and 6, respectively.

For Italy, the overall sensitivity for EVI was 0.82 (95% Confidence Intervals: 0.75; 0.89) and the specificity was 0.91 (0.88; 0.94). For New York, the corresponding values were 0.55 (0.47; 0.64) and 0.88 (0.84; 0.91).

**Figure 7:**
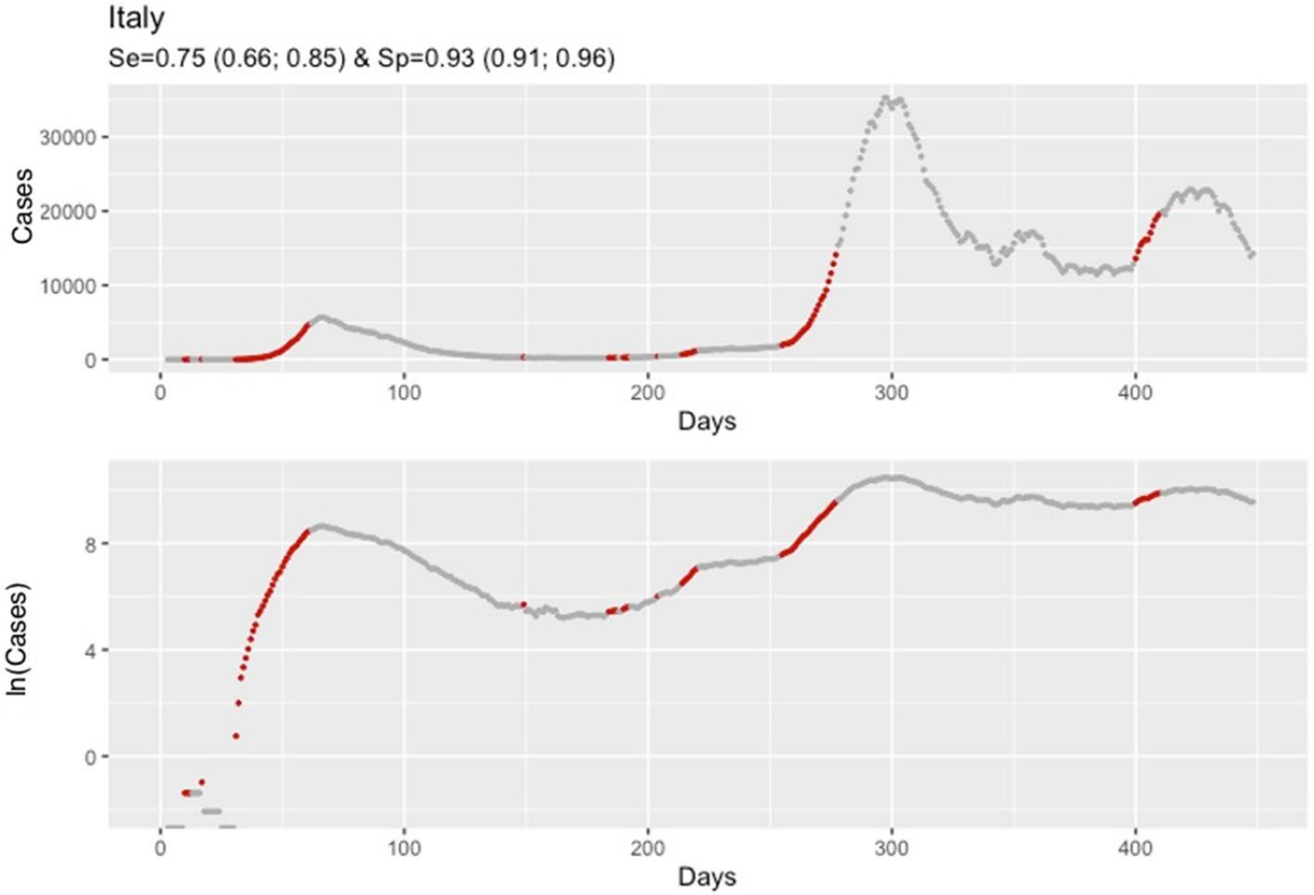
Daily confirmed cases of COVID-19 in Italy, from January 22, 2020, until April 13, 2021. Analysis is based on the criterion aiming to detect an increase in the mean of expected cases equal or higher than 50%. Red dots correspond to dates that, based on EVI, an early warning was issued indicating that a rise in the COVID-19 cases is expected. Data are presented on the original scale (**1a**) and the logarithmic scale (**1b**) which facilitates the comparison of the steepness of the epidemic curve between the different waves.

Sensitivity analysis results for Italy are in Fig. 7. Under the alternative criterion aiming to detect an increase in the mean of expected cases equal or higher than 50%, the overall sensitivity and specificity were 0.75 (0.66; 0.85) and 0.93 (0.91; 0.96), respectively.

A consistent finding in the results from all countries was that consecutive early warnings are linked to the start of a new epidemic wave, while the absence of warnings indicates a stable course or a future drop in the number of new COVID-19 cases (Fig. 3, 4 and http://83.212.174.99:3838/).

## Discussion

EVI is a useful and easy to implement early-warning tool for an upcoming rise in the number of new cases. Results revealed a reliable ability of EVI to predict the COVID-19 epidemic waves, in all instances, as expressed by its overall $Se$ and $Sp$. A more important aspect lies in the fact that repetitive issuance of early warnings indicates the beginning of an epidemic wave. This is a consistent and stable finding across all countries and each of the United States (Figs. 3, 4 and http://83.212.174.99:3838/). In a similar manner, the absence of a series of early warnings implies that the number of new cases will remain stable or drop. The latter was also a consistent finding. Additionally, false early warnings (i.e. false positives) were isolated instances and did not occur in a consecutive series. There were few occasions with a consecutive absence of early warnings despite a continuing rise in the number of cases (i.e. false negatives). Nevertheless, such series of false negatives were always close to the peak of a wave. This finding is reasonable and could be interpreted as an early sign of reaching the peak because EVI depends on the volatility and the increase in the number of new cases decelerates when approaching the peak of an epidemic wave. Positive and negative predictive values, which are calculated at each time point, can also be used to assess the probability that an early warning, or its absence, is true. In all instances, predictive values were high with the exception of few instances at the beginning of the time series where there were insufficient data.

Previous work on compartmental SIR and SIS models has revealed that moving window estimates of the variance increase while approaching the emergence of a pathogen as well as during the elimination phase and that it can be used as an early warning tool^25^. EVI is based on the relative rather than the absolute change of the standard deviation because the latter depends on the underlying prevalence at each time point of the epidemic. Hence, a low threshold would be efficient in detecting a surge in the new cases at the beginning of an epidemic, when the baseline prevalence is low, but would have failed to do so for subsequent epidemic waves that commence from a higher baseline prevalence. On the other hand, a high absolute threshold would have failed to capture waves at the beginning of the epidemic. EVI is based on the relative increase in volatility, which implicitly adjusts for the baseline prevalence at each point of the time series.

In general, the ability of EVI to provide valid predictions does not seem to be affected by the fact that sampling and testing schemes for COVID-19 are mainly based on passive surveillance systems. EVI performed equally well among different countries with different control strategies, testing intensity and reporting accuracy and despite the fact that, even within countries, sampling and testing has changed over time and/or differs between regions^26,27^. Restriction of the maximum window size $\left( {m_{{{\text{max}}}} } \right)$ to one month plays a key role, because reporting bias is expected to remain similar over short time periods. This form of non-differential misclassification leads to reporting rates that, though biased, do not have a significant impact on volatility, EVI and its predictive ability. Crucially, it is important that the data do not exhibit strong artifacts of recording bias, as there is no way for the method to distinguish between a trend due to underlying epidemic patterns and an observed trend due to changes in reporting practices or an increased testing capacity or effort^28^. This could for instance happen when a country changes its general testing regime, experiences local outbreaks, and focuses testing on a specific area or targets other subgroups of the population than previously. Thus, EVI should preferably be evaluated for use in smaller geographical regions, such as counties or municipalities, if sufficient, high-quality data is available. Undoubtedly, all models are prone to limitations due to imperfect data^9^ but the continuing enhancement of active and passive surveillance systems—as the testing regimes and methods also improve—will lead to improved data quality.

The performance of EVI depends on the specified criterion which should be epidemic-specific and can be country-specific. Modifications to allow for an alternative criterion, for the different periods of an epidemic, are rather straightforward to implement. Parameters $c$ and $m$ are allowed to vary and take values that would satisfy the conditions set by the defined criterion and the desired accuracy. A point of concern is the selection of the maximum window size $m_{{{\text{max}}}}$. For an ongoing epidemic with multiple waves, as is the case with COVID-19, $m_{{{\text{max}}}}$ should be limited to a period shorter than the entire observation period. This prevents excess volatility of past epidemic waves from affecting the most recent volatility estimates and the ability of EVI to warn for upcoming waves that may be smaller and of lower volatility than previous ones. In our example, we limited $m_{{{\text{max}}}}$ to one month. EVI also depends on data intensity. Detailed data at the lowest time unit (i.e., days rather than weeks) is preferable in order to detect changes rapidly. In the COVID-19 example the 7-day moving average was analyzed instead of the daily reported cases because daily data had unnatural variability due to reporting variations between working days and weekends. Nevertheless, analysis based on the daily reported cases provided similar results (data not shown here).

Beyond the case of epidemics or exceptional events, like the COVID-19 pandemic, an important application of EVI could be in the context of syndromic surveillance^29^, not limited to outbreaks from biologic terrorism, but in its broader sense: the detection of temporal and spatial aberrations in the expected number of cases for signs and symptoms. Such systems already exist and utilize state-of-the-art information technologies within the context of public health^1^ as well as one health^30,31^. EVI could provide an additional early warning tool in support of these systems.

### Supplementary Information

Supplementary Information.

## Data Availability

Daily updated results/predictions are available at http://83.212.174.99:3838.

## Appendix

### The epidemic volatility index

EVI is calculated for a rolling window of time series epidemic data (i.e. the number of new cases per day). At each step, the observations within the window are obtained by shifting the window forward over the time series data one observation at a time.

Let $x_{i} = \left\{ {x_{1} ,x_{2} , \ldots ,x_{n} } \right\}$ be a time series of length $N$. The rolling window size—that is the number of consecutive observations per rolling window − is $m$. With $0 < m \le m_{{{\text{max}}}}$ and $0 < m_{{{\text{max}}}} \le N$, there are  $t = N - m + 1$ consecutive rolling windows.

At each of the $t$ steps, $EVI$ uses the standard deviation $\left( {s_{t} } \right)$ of the newly reported cases $\left( {y_{{j_{t} }} = \left\{ {y_{{1_{t} }} ,y_{{2_{t} }} , \ldots ,y_{{m_{t} }} } \right\}} \right)$ within the specified $m$

$$s_{t} = \sqrt {\frac{1}{m}\mathop \sum \limits_{{i_{t} = 1}}^{m} \left( {x_{{i_{t} }} - \overline{x}_{t} } \right)^{2} }$$

with $\overline{{x_{t} }}$ the mean of the *t*th window. Subsequently, EVI is calculated as the relative change of $\left( {s_{t} } \right)$ between two consecutive rolling windows:

$$EVI_{t - 1,t} = \frac{{s_{t} - s_{t - 1} }}{{s_{t - 1} }}$$

We expect an increase in the future number of cases, if $EVI_{t - 1,t}$ exceeds a threshold $c$ $\left( {c \in \left[ {0,1} \right]} \right)$ and the observed cases at time point $t,\left( {y_{t} } \right)$ are higher than the average of the reported cases in the previous week:

$$Ind_{{EVI_{t - 1,t} }} = \left\{ {\begin{array}{*{20}l} 1 \hfill & {if\;EVI_{t - 1,t} \ge c \wedge y_{t} \ge \overline{\mu }_{t:t - 7} } \hfill \\ 0 \hfill & {{\text{otherwise}}} \hfill \\ \end{array} } \right.$$

### Criterion and desired accuracy

The user should provide the minimum rise in cases that, if present, should be detected. A criterion can be the rise in the mean number of cases between two consecutive weeks that exceeds a threshold:

$$\frac{{\overline{\mu }_{t:t + 7} - \overline{ \mu }_{t:t - 7} }}{{\overline{\mu }_{t:t - 7} }} \ge r$$

with $$0 \le r \le 1$$.

The accuracy of EVI, given the specified criterion, depends on $m$ and $c$, which should be selected in a way to achieve a desired accuracy target. Several strategies are available. One option is the selection of *m* and *c* values that lead to the best *Se* and *Sp* combination for EVI, through the maximization of the Youden index $\left( {J = Se + Sp - 1} \right)$^12^ and, hence, the overall minimization of false results (i.e., the total number of false positive and false negative early warnings). Another approach could be to select $m$ and $c$. such that the highest $Se \left( {or\;Sp} \right)$ is achieved with $Sp \left( {or\;Se} \right) = 1$ or not dropping below a critical value (e.g. 0.95). Advanced Receiver Operating Characteristic curve analysis can also be performed^13^ and selection of critical values can be based on indices that quantify the relative cost of false positive (i.e., falsely predicting an upcoming epidemic wave) to false negative (i.e., failing to predict an upcoming epidemic wave) warnings, like the misclassification cost term $\left( {MCT} \right)$.

### Generation of an early warning

Every time a new time point $t$ is observed, the model uses all the observed cases up to $t$ to decide whether it should issue an early warning, at time point $t$. The steps are:

1. Observed cases up to $t$ are analyzed for all possible values of the window size $\left( {m \in \left[ {1,m_{{{\text{max}}}} } \right]} \right)$ and threshold $\left( {c \in \left[ {0,1} \right]} \right)$.
2. For each of the *m* and *c* combinations, the $Se_{{t_{m,c} }}$ and $Sp_{{t_{m,c} }}$ are estimated for the predefined criterion (Eq. 4).
3. The $m^{{\prime }}$ and $c^{{\prime }}$ that give the best $Se_{{t_{m^{\prime},c^{\prime}} }}$ and $Sp_{{t_{m^{\prime},c^{\prime}} }}$ combination are selected.
4. For $m^{{\prime }}$ and $c^{{\prime }}$, the value of $Ind_{{EVI_{t,t - 1} }}$ is determined at the most recent time point *t* and a decision is made on whether or not a warning signal is issued.

### Accuracy and predictive values

Further, at each time point $t$, the probability of observing a rise or drop in the future cases, given that an early warning was issued or not, can be calculated as the positive $\left( {PV_{t} + } \right)$ and negative $\left( {PV_{t} - } \right)$ predictive value, respectively:

$$PV_{t} + = P\left( {D + {\mid }T + } \right) = \frac{{p_{1:t} Se_{{t_{m^{\prime},c^{\prime}} }} }}{{p_{1:t} Se_{{t_{m^{\prime},c^{\prime}} }} + \left( {1 - p_{1:t} } \right)\left( {1 - Sp_{{t_{m^{\prime},c^{\prime}} }} } \right)}}$$

$$PV_{t} - = P\left( {D - {\mid }T - } \right) = \frac{{\left( {1 - p_{1:t} } \right)Sp_{{t_{m^{\prime},c^{\prime}} }} }}{{\left( {1 - p_{1:t} } \right)Sp_{{t_{m^{\prime},c^{\prime}} }} + p_{1:t} \left( {1 - Se_{{t_{m^{\prime},c^{\prime}} }} } \right)}}$$

where $p_{1:t}$ is the proportion of events satisfying the condition of Eq. 4 up to time point $t$.

Once the entire time series data have been observed, the overall $Se_{EVI}$ can be estimated as the fraction of the total number of occurrences for which an early warning has been issued, given that the criterion (Eq. 4 ) holds $\left( {P\left( {T + {\mid }D + } \right)} \right)$, divided by the total number of occurrences that the criterion holds $\left( {P\left( {D + } \right)} \right)$. Similarly, the overall $Sp_{EVI}$ is calculated as the fraction of the total number of occurrences for which an early warning was not issued given that the expected rise of cases was not observed, that is, the criterion is not true, $\left( {P\left( {T - {\mid }D - } \right)} \right)$ divided by the total number of occurrences that the criterion is not true $\left( {P\left( {D - } \right)} \right)$:

$$Se_{EVI} = \frac{{P\left( {T + {\mid }D + } \right)}}{{P\left( {D + } \right)}},\;Sp_{EVI} = \frac{{P\left( {T - {\mid }D - } \right)}}{{P\left( {D - } \right)}}$$

### Sensitivity analysis

The performance of EVI depends on the specified criterion (i.e., $r$) and the desired accuracy. Ideally, in the presence of historical data, various criterion values ($r$ values) should be explored to identify combinations that provide the optimal monitoring of an epidemic.

## References

[1] Heffernan R, Mostashari F, Das D, Karpati A, Kulldorff M, Weiss D (2004). Syndromic Surveillance in Public Health Practice.

[2] Brett TS, Rohani P (2020). Dynamical footprints enable detection of disease emergence. PLoS Biol..

[3] Vega T, Lozano JE, Meerhoff T, Snacken R, Mott J, Ortiz de Lejarazu R, Nunes B (2013). Influenza surveillance in Europe: establishing epidemic thresholds by the moving epidemic method. Influenza Other Respir. Viruses.

[4] Abeku TA, Hay SI, Ochola S, Langi P, Beard B, de Vlas SJ, Cox J (2004). Malaria epidemic early warning and detection in African highlands. Trends Parasitol..

[5] Chang F-S, Tseng Y-T, Hsu P-S, Chen C-D, Lian I-B, Chao D-Y (2015). Re-assess vector indices threshold as an early warning tool for predicting dengue epidemic in a dengue non-endemic country. PLoS Negl. Trop. Dis..

[6] Chowell G, Sattenspiel L, Bansal S, Viboud C (2016). Mathematical models to characterize early epidemic growth: a review. Phys. Life Rev..

[7] Wang P, Zheng X, Li J, Zhu B (2020). Prediction of epidemic trends in COVID-19 with logistic model and machine learning technics. Chaos Solitons Fractals.

[8] Kogan NE, Clemente L, Liautaud P, Kaashoek J, Link NB, Nguyen AT, Lu FS, Huybers P, Resch B, Havas C, Petutschnig A, Davis J, Chinazzi M, Mustafa B, Hanage WP, Vespignani A, Santillana M (2021). An early warning approach to monitor COVID-19 activity with multiple digital traces in near real time. Sci. Adv..

[9] Vespignani A, Tian H, Dye C, Lloyd-Smith JO, Eggo RM, Shrestha M, Scarpino SV, Gutierrez B, Kraemer MUG, Wu J, Leung K, Leung GM (2020). Modelling COVID-19. Nat. Rev. Phys..

[10] Brenner M, Galai D (1989). New financial instruments for hedge changes in volatility. Financ. Anal. J..

[11] Fernandes M, Medeiros MC, Scharth M (2014). Modeling and predicting the CBOE market volatility index. J. Bank. Finance.

[12] Fluss R, Faraggi D, Reiser B (2005). Estimation of the Youden index and its associated cutoff point. Biom. J..

[13] Zweig MH, Campbell G (1993). Receiver-operating characteristic (ROC) plots: a fundamental evaluation tool in clinical medicine. Clin. Chem..

[14] Fauci AS, Lane HC, Redfield RR (2020). Covid-19 navigating the uncharted. N. Engl. J. Med..

[15] Ciotti M, Ciccozzi M, Terrinoni A, Jiang W-C, Wang C-B, Bernardini S (2020). The COVID-19 pandemic. Critical Reviews in Clinical Laboratory Sciences.

[16] Dong E, Du H, Gardner L (2020). An interactive web-based dashboard to track COVID-19 in real time. Lancet Infect. Dis..

[17] R Core Team, R: A language and environment for statistical computing. (2020).

[18] Wickham, H. *et al.* Package ‘readxl’. (2019).

[19] Wickham H (2011). ggplot2. Comput. Stat..

[20] Wilke, C.O., Wickham, H., Wilke, M.C.O. Package ‘cowplot’. Streamlined Plot Theme and Plot Annotations for ‘ggplot2. (2019).

[21] Wickham, H. *et al.* Package ‘readr’. (2015).

[22] Furuya-Kanamori, L., & Kostoulas, P. EVI: Stata module to compute Epidemic Volatility Index (EVI) for detecting epidemic waves. https://EconPapers.repec.org/RePEc:boc:bocode:s459005 (2021).

[23] Livingston E, Bucher K (2020). Coronavirus disease 2019 (COVID-19) in Italy. JAMA.

[24] Thompson CN, Baumgartner J, Pichardo C, Toro B, Li L, Arciuolo R, Chan PY, Chen J, Culp G, Davidson A (2020). COVID-19 outbreak—New York City, February 29–June 1, 2020. Morb. Mortal. Wkly. Rep..

[25] O’Regan SM, Drake JM (2013). Theory of early warning signals of disease emergenceand leading indicators of elimination. Thyroid Res..

[26] Brynildsrud O (2020). COVID-19 prevalence estimation by random sampling in population-optimal sample pooling under varying assumptions about true prevalence. BMC Med. Res. Methodol..

[27] Middelburg RA, Rosendaal FR (2020). COVID-19: How to make between-country comparisons. Int. J. Infect. Dis..

[28] Halasa T, Græsbøll K, Denwood M, Christensen LE, Kirkeby C (2020). Prediction models in veterinary and human epidemiology: our experience with modeling sars-CoV-2 spread. Front. Vet. Sci..

[29] Henning KJ (2004). What is syndromic surveillance?. Morb. Mortal. Wkly. Rep..

[30] Beltrán-Alcrudo D, Carpenter TE, Cardona C (2009). A flock-tailored early warning system for low pathogenic avian influenza (LPAI) in commercial egg laying flocks. Prev. Vet. Med..

[31] Gilbert M, Golding N, Zhou H, Wint GR, Robinson TP, Tatem AJ, Lai S, Zhou S, Jiang H, Guo D, Huang Z, Messina JP, Xiao X, Linard C, Van BTP, Martin V, Bhatt S, Gething PW, Farrar JJ, Hay SI, Yu H (2014). Predicting the risk of avian influenza A H7N9 infection in live-poultry markets across Asia. Nat. Commun..

