# Supplementary material for: The epidemic volatility index, a novel early warning tool for identifying new waves in an epidemic
Source: Sci Rep. 2021 Dec 10;11:23775. doi: 10.1038/s41598-021-02622-3 (PMC8664819; doi:10.1038/s41598-021-02622-3)
Supplement: Supplementary file 1 — Supplementary Information. [file 41598_2021_2622_MOESM1_ESM.docx]

# Appendix

## The Epidemic Volatility Index

    EVI is calculated for a rolling window of time series epidemic data (i.e. the number of new cases per day). At each step, the observations within the window are obtained by shifting the window forward over the time series data one observation at a time.

Let $x_{i}=\left\{ x_{1}, x_{2},...,x_{n} \right\}$ be a time series of length $N$. The rolling window size - that is the number of consecutive observations per rolling window - is $m$. With $0<m\leq m_{\max}$ and $0<m_{\max}\leq N$, there are  $t=N-m+1$ consecutive rolling windows.

    At each of the $t$ steps, $EVI$ uses the standard deviation $\left( s_{t} \right)$ of the newly reported cases $\left( y_{j_{t}}=\left\{ y_{1_{t}}, y_{2_{t}},... ,y_{m_{t}} \right\} \right)$ within the specified $m$

$$s_{t}=\sqrt{\frac{1}{m}\sum_{i_{t}=1}^{m} \left( x_{i_{t}}-\overline{x}_{t} \right)^{2}}$$

with $\overline{x_{t}}$ the mean of the $t^{th}$ window. Subsequently, EVI is calculated as the relative change of $\left( s_{t} \right)$ between two consecutive rolling windows:

$$EVI_{t-1,t}=\frac{s_{t}-s_{t-1}}{s_{t-1}}$$

    We expect an increase in the future number of cases, if $EVI_{t-1,t}$ exceeds a threshold $c$ $\left( c\in\left[ 0,1 \right] \right)$ and the observed cases at time point $t,\left( y_{t} \right)$ are higher than the average of the reported cases in the previous week:

$$Ind_{EVI_{t-1,t}}=\left\{ \begin{matrix} 1 if EVI_{t-1,t}\geq c \wedge y_{t}\geq\overline{\mu}_{t:t-7} \\ 0 \mathrm{otherwise} \end{matrix} \right.$$

## Criterion and desired accuracy

    The user should provide the minimum rise in cases that, if present, should be detected. A criterion can be the rise in the mean number of cases between two consecutive weeks that exceeds a threshold:

$$\frac{\overline{\mu}_{t:t+7}-\overline{\mu}_{t:t-7}}{\overline{\mu}_{t:t-7}}\geq r$$

with $0\leq r\leq1$.

    The accuracy of EVI, given the specified criterion, depends on $m$ and $c$, which should be selected in a way to achieve a desired accuracy target. Several strategies are available. One option is the selection of *m* and *c* values that lead to the best *Se* and *Sp* combination for EVI, through the maximization of the Youden index $\left( J=Se+Sp-1 \right)$ (Fluss et al., 2005) and, hence, the overall minimization of false results (i.e., the total number of false positive and false negative early warnings). Another approach could be to select $m$ and $c$ such that the highest $Se \left( or Sp \right)$ is achieved with $Sp \left( or Se \right)=1$ or not dropping below a critical value (e.g. 0.95). Advanced Receiver Operating Characteristic curve analysis can also be performed (Zweig and Campbell, 1993) and selection of critical values can be based on indices that quantify the relative cost of false positive (i.e., falsely predicting an upcoming epidemic wave) to false negative (i.e., failing to predict an upcoming epidemic wave) warnings, like the misclassification cost term $\left( MCT \right)$.

## Generation of an early warning

    Every time a new time point $t$ is observed, the model uses all the observed cases up to $t$ to decide whether it should issue an early warning, at time point $t$. The steps are:

1. Observed cases up to $t$ are analyzed for all possible values of the window size $\left( m\in\left[ 1,m_{\max} \right] \right)$ and threshold $\left( c\in\left[ 0,1 \right] \right)$.
2. For each of the $m and c$ combinations, the $Se_{t_{m,c}}$and $Sp_{t_{m,c}}$are estimated for the predefined criterion (Eq. 4).
3. The  $m'$ and $c'$ that give the best $Se_{t_{m',c'}}$ and $Sp_{t_{m',c'}}$ combination are selected.
4. For $m'$ and $c'$, the value of $Ind_{EVI_{t,t-1}}$ is determined at the most recent time point $t$ and a decision is made on whether or not a warning signal is issued.

## Accuracy and Predictive Values

    Further, at each time point $t$, the probability of observing a rise or drop in the future cases, given that an early warning was issued or not, can be calculated as the positive $\left( PV_{t}+ \right)$ and negative $\left( PV_{t}- \right)$ predictive value, respectively:

$$PV_{t}+=P(D+\mid T+)=\frac{p_{1:t}Se_{t_{m',c'}}}{p_{1:t}Se_{t_{m',c'}}+\left( 1-p_{1:t} \right)\left( 1-Sp_{t_{m',c'}} \right)}$$

$$PV_{t}-=P(D-\mid T-)=\frac{\left( 1-p_{1:t} \right)Sp_{t_{m',c'}}}{\left( 1-p_{1:t} \right)Sp_{t_{m',c'}}+p_{1:t}\left( 1-Se_{t_{m',c'}} \right)}$$

where $p_{1:t}$ is the proportion of events satisfying the condition of Eq. 4 up to time point $t$.

Once the entire time series data have been observed, the overall $Se_{EVI}$ can be estimated as the fraction of the total number of occurrences for which an early warning has been issued, given that the criterion (Eq. 4 ) holds $(P(T+\mid D+))$, divided by the total number of occurrences that the criterion holds $(P(D+))$. Similarly, the overall $Sp_{EVI}$ is calculated as the fraction of the total number of occurrences for which an early warning was not issued given that the expected rise of cases was not observed, that is, the criterion is not true, $(P(T-\mid D-))$ divided by the total number of occurrences that the criterion is not true $\left( P\left( D- \right) \right)$:

$$Se_{EVI}=\frac{P\left( T+\mid D+ \right)}{P\left( D+ \right)}, Sp_{EVI}=\frac{P\left( T-\mid D- \right)}{P\left( D- \right)}$$

## Sensitivity analysis

    The performance of EVI depends on the specified criterion (i.e., $r$) and the desired accuracy. Ideally, in the presence of historical data, various criterion values ($r$ values) should be explored to identify combinations that provide the optimal monitoring of an epidemic.
